# Vanadium-protein complex inhibits human adipocyte differentiation through the activation of β-catenin and LKB1/AMPK signaling pathway

**DOI:** 10.1371/journal.pone.0239547

**Published:** 2020-09-24

**Authors:** Shuang Zhang, Lei Yan, Sang Moo Kim

**Affiliations:** 1 Heilongjiang Provincial Key Laboratory of Environmental Microbiology and Recycling of Argo-Waste in Cold Region, College of Life Science and Biotechnology, Heilongjiang Bayi Agricultural University, Daqing, Heilongjiang Province, People’s Republic of China; 2 Department of Marine Food Science and Technology, Gangneung-Wonju National University, Gangneung, Gangwon-do, Republic of Korea; Tohoku University, JAPAN

## Abstract

Obesity is a common disease over the world and is tightly associated with diabetes mellitus, cardiovascular and cancer disease. Although our previous study showed that the synthetic vanadium-protein (V-P) complex had a better effect on antioxidant and antidiabetic, the relative molecular mechanisms are still entirely unknown. Hence, we investigated the effect of the synthetic V-P complex on adipocyte differentiation (adipogenesis) using human preadipocytes to clarify its molecular mechanisms of action. The primary human preadipocytes were cultured with and without V-P complex during adipocyte differentiation. The cell proliferation, lipid accumulation, and the protein expression of transcription factors and related enzymes were determined for the differentiated human preadipocytes. In this study, the 20 μg/mL of V-P complex reduced the lipid and triglyceride (TG) content by 74.47 and 57.39% (p < 0.05), respectively, and down-regulated the protein expressions of peroxisome proliferator-activated receptor-γ (PPARγ), CCAAT/enhancer-binding protein alpha (C/EBPα), sterol regulatory element-binding protein 1 (SREBP-1) and fatty acid synthase (FAS). Additionally, the V-P complex significantly up-regulated the protein levels of total β-catenin (t-β-catenin), nuclear β-catenin (n-β-catenin), phosphorylated adenosine monophosphate-activated protein kinase alpha (p-AMPKα) and liver kinase B1 (p-LKB1). These showed that the inhibitory effect of V-P complex on human adipogenesis was mediated by activating Wnt/β-catenin and LKB1/AMPK-dependent signaling pathway. Therefore, the synthetic V-P complex could be considered as a candidate for prevention and treatment of obesity.

## Introduction

Obesity is not only a disease between adult males and females but also a worldwide epidemic [1, 2]. It is associated with inherited background and living environments including socioeconomic status, living and eating habits [3]. Obesity can cause health problems such as type-2 diabetes, cardiovascular, hyperlipidemia, hypercholesterolemia, hypertension and cancer disease [1, 4–6]. It has been reported that more than 600 million adults are obesity or weight gain around the world in 2015 [7]. A large number of studies were carried out in human and non-human models of obesity, indicating that the increase in cell size (hypertrophy) and number (hyperplasia) of adipocytes play a crucial role in obesity [3]. Adipocytes are highly specialized cells and can be generated from inducing preadipocytes in the involvement of a series of adipogenic transcription factors and enzymes [8]. It has been demonstrated that several adipogenic transcription factors including PPARγ, C/EBP family (C/EBPα, β, γ), and SREBP-1c can induce adipocyte differentiation (adipogenesis) [7]. Among them, PPARγ, considered as a master regulator for adipocyte differentiation, mainly expressed in adipose tissue and is both necessary and sufficient for adipocyte differentiation [9, 10]. The roles of C/EBP family members in adipogenesis were also interrupted. The activation of C/EBPα was found to be beneficial for enhancing insulin sensitivity in adipocytes [9], and the activation of C/EBPβ and C/EBPγ can stimulate the expression of PPARγ and C/EBPα [8, 11], which can further regulate the expression of SREBP-1c and downstream adipocyte-specific proteins involved in lipolysis [7].

It has been demonstrated that many pathways such as Wnt/β-catenin, and LKB1/AMPK signaling pathway involved in the regulation of adipocyte differentiation through the regulation of transcription factors PPARγ and C/EBPα [10, 12]. In the Wnt/β-catenin signaling, Wnt can affect the adipocyte differentiation based on the β-catenin-dependent (canonical Wnt) and β-catenin-independent (non-canonical Wnt) mechanisms, in which β-catenin plays a central role in inhibiting adipogenesis [9]. LKB1 and AMPK are considered to be essential mediators in both adipogenesis and lipolysis. AMPK, a primary downstream signal for LKB1 [13], is a cellular energy sensor that can regulate glucose and lipid metabolism by monitoring the change in AMP/ATP ratio [10]. AMPK activation can inhibit the expression of downstream adipocyte differentiation transcription factors including PPARγ, C/EBPα, SREBP-1. Whereafter these transcription factors can regulate the expression of lipogenic genes including acetyl-CoA carboxylase (ACC), FAS and fatty acid-binding protein 4 (FABP4). These would lead to inhibition of adipocyte differentiation and further antiobesity [7, 14]. Therefore, the inhibition of adipocyte differentiation was regarded as a rational treatment strategy for obesity. In recent decades, some natural bioactive compounds such as ursolic acid, sulfated glucosamine, quercetin, fucoidan and ginsenoside have been reported to inhibit adipocyte differentiation by activating Wnt/β-catenin and LKB1/AMPK pathway [4, 12, 14–16]. However, several shortcomings including low extraction efficiency, high cost and time-consuming of these extracted natural compounds limited their clinical and commercial applications.

Therefore, scholars began to pay attention to synthetic various bioactive compounds that possessed antiobesity activity. It has been reported that the synthetic compounds including 1,2-naphthoquinone derivatives, cyano-pyrazoline derivatives and metal-based (V, Cr, Mo, Zn, Cu and Mn) compounds showed distinct antidiabetic activity [17–19]. Among them, the metal-based compounds had attracted more interest due to their diverse and unpredictable biological activities [19, 20]. Vanadium, a transition metal, has been attracted much attention due to its insulin-mimetic actions and antidiabetic effects *in vivo* and *in intro*. Inorganic vanadium compounds including ammonium metavanadate (AMV) and sodium orthovanadate showed insulin-like action in female wistar rats [21, 22]. Organic vanadium compounds such as vanadium-flavonol complex could reduce blood glucose in STZ-induced rats [2]. Natural vanadium compounds such as vanadium-binding protein (VBP) extracted from sea cucumber or sea squirt could inhibit adipocyte differentiation in 3T3-L1 cells [12, 23]. Additionally, our previous study showed that the synthetic vanadium-protein complex had antidiabetic activity *in vitro* [24]. It has been demonstrated that vanadium complexes with proper organic ligands can enhance vanadium’s liposoluble properties, promote gastro-intestinal absorption, tissue uptake and reduce the toxicity of the vanadium [25]. However, the underlying molecular mechanism of organic vanadium compounds (V-P complex) has not yet been elucidated.

Therefore, in the present study, we evaluated the effect of the synthetic V-P complex on adipogenesis and protein expression of adipocyte differentiation transcription factors. Furthermore, the Wnt/β-catenin and LKB1/AMPK signaling pathway and their related targets were investigated in primary human preadipocytes.

## Materials and methods

### Materials

Recombinant human sentrin-specific protease 8 (N-6His) was obtained from Novoprotein (Shanghai, China). All antibodies were purchased from Cell Signaling Technology Inc. (Danvers, MA, USA). Oil Red O and triglyceride assay kit were purchased from Sigma-Aldrich (St. Louis, MO, USA). WST-1 solution [2-(4-nitrophenyl)-5-(2-sulfophenyl)-3-[4-(4-sulfophenylazo)-2-sulfophenyl]-2Htetrazolium disodium salt] (EZ-cytox assay kit) was purchased from DoGenBio Co., Ltd (Seoul, Korea), 4% Paraformaldehyde (PFA) in PBS was purchased from Thermo Fisher Scientific (Waltham, MA, USA). Preadipocyte medium (PM) and adipocyte differentiation medium (DM) were purchased from Zenbio (Research Triangle Park, NC, USA). Vandal sulfate (VS) was purchased from Daejung Chemical & Materials Co. Ltd (Gyeonggi-do, Korea).

### Synthesis of V-P complex

The V-P complex was synthesized according to the method of Zhang and Kim [24]. Briefly, 500 μg of recombinant human sentrin-specific protease 8 (N-6His) (26.2 kDa) was loaded to a Sephacryl S-200 HR column (1.0 × 10.0 cm) equilibrated with a binding buffer (1 mM VS, 1 mM iminodiacetic acid, 100 mM NaCl, 10 mM Tris, pH 7.4), and then eluted with binding buffer at a flow rate of 0.3 mL/min. Thereafter, the eluted fractions were dialyzed against 50 mM Tris-HCl buffer (pH 7.4) to remove unbound vanadium and then concentrated by ultrafiltration (cut-off 10 kDa) at 2000×g (Centrifuge 5810 R; Eppendorf AG, Hamburg, Germany) at 4°C. The concentration of vanadium was determined on a optima 8300 inductively coupled plasma optical emission spectrometry (ICP-OES) (Perkin Elmer, Waltham, MA, USA) according to the references [26, 27].

### Cell culture and differentiation

Cryopreserved primary human preadipocytes (Item # SP-F-SL) were purchased from Zenbio (Research Triangle Park, NC, USA), which derived from subcutaneous adipose tissues of five female donors with an average age of 47.2 years and average body mass index of 28.1 kg/m^2^. Human preadipocytes were grown in PM at 37 ^o^C in a humidified 5% CO_2_ atmosphere (New Brunswich ^TM^ Galaxy 170S; Eppendorf AG, Hamburg, USA). Upon reaching confluence, the culture media were replaced with DM and considered as Day “0”. Subsequently, the culture media were changed every two days until the cells were harvested. Human preadipocytes were treated with V-P complex during differentiation from day 0 until the cells were harvested for analysis.

### Cell viability

Cell viability was assayed with the EZ-cytox assay kit according to the manufacturer’s instruction. Human preadipocytes (4 × 10^4^ cells/ mL) were seeded into 96-well plates and treated at day 0 with different concentrations of V-P complex (5, 10, 20, 40 μg/mL). After culturing for 5, 10, 14 days, 10 μL of WST was added to each well and then the cells were incubated at 37 ^o^C for 4 h. The absorbance at 450 nm was measured using a microplate reader (EL-800, BioTek, Winooski, VT, USA). The viability (%) was calculated by the following equation:

$$Viability\;(\%)=\frac{As-Ab}{Ac-Ab}\times 100$$ (1)

where As, Ab and Ac are the absorbances of the sample, negative control (without cells) and positive control (without sample), respectively.

### Oil red O staining

Oil red O staining was performed according to the method of Nerukar et al. [28]. The differentiated human preadipocytes were fixed at room temperature overnight with 4% PFA after washing with cold PBS twice, followed by washing twice with PBS and staining with freshly 0.3% oil red O at room temperature for 15 min. Stained lipid droplets were extracted in isopropanol and quantified by measuring the absorbance at 490 nm using a microplate reader (BioTek). The percentage of adipogenesis was calculated by the following equation:

$$Adipogenesis\;(\%)=\frac{A_{t}}{A_{u}}\times 100$$ (2)

where At and Au are the absorbances of the treated human preadipocytes and untreated human preadipocytes, respectively.

### Cellular triglyceride analysis

Human preadipocytes were treated with different concentrations of V-P complex (5, 10, 20 μg/mL) in 96-well plates during differentiation. Cellular TG content was measured with the triglyceride assay kit according to the manufacturer’s instruction. The TG content was calculated by the following equation:

C=Sa/Sv (3)

where C is the concentration of TG in the sample, Sa is the amount of TG in sample and Sv is the sample volume added to reaction well.

### Western blot analysis

Total proteins were extracted from the human preadipocytes treated with V-P complex at day 14. The differentiated human preadipocytes were washed with cold PBS and lysed in RIPA lysis buffer supplemented with protease and phosphatase inhibitor (T&I, Seoul, Korea). Protein samples (25 μg/lane) were separated by 10% sodium dodecyl sulfate-polyacrylamide gel electrophoresis (SDS-PAGE) and transferred to polyvinylidene fluoride (PVDF) membranes. The PVDF membranes were blocked at room temperature for 1 h in 5% non-fat skim milk, and then incubated with the primary antibodies (diluted 1:2000) in tris-buffered saline with Tween-20 (TBST) at 4 ^o^C overnight, followed by incubating with the secondary antibody (1:2000) (horseradish-peroxidase-conjugated anti-rabbit antibody) at room temperature for 1 h. The immune reactive bands were visualized by ECL reagents (Thermo Fisher Scientific, Waltham, MA, USA). Imaging and densitometry were analyzed using a ChemiDoc XRS+ System with Image Lab Software (Bio-Rad Laboratories, Hercules, CA, USA).

### Statistical analysis

All experiments were performed in triplicate, and the data were presented as mean values ± SD. A one-way analysis of variance (ANOVA) and Duncan’s test with a significant threshold set at 0.05 (p < 0.05) were used to evaluate the differences using SPASS software (SPASS 19.0; SPSS company, Chicago, IL, USA). All figures were made using GraphPad Prism version 7.0 for Mac (GraphPad Software Inc., San Diego, CA, USA).

## Results and discussion

### Effect of V-P complex on cell viability

**Fig 1:**
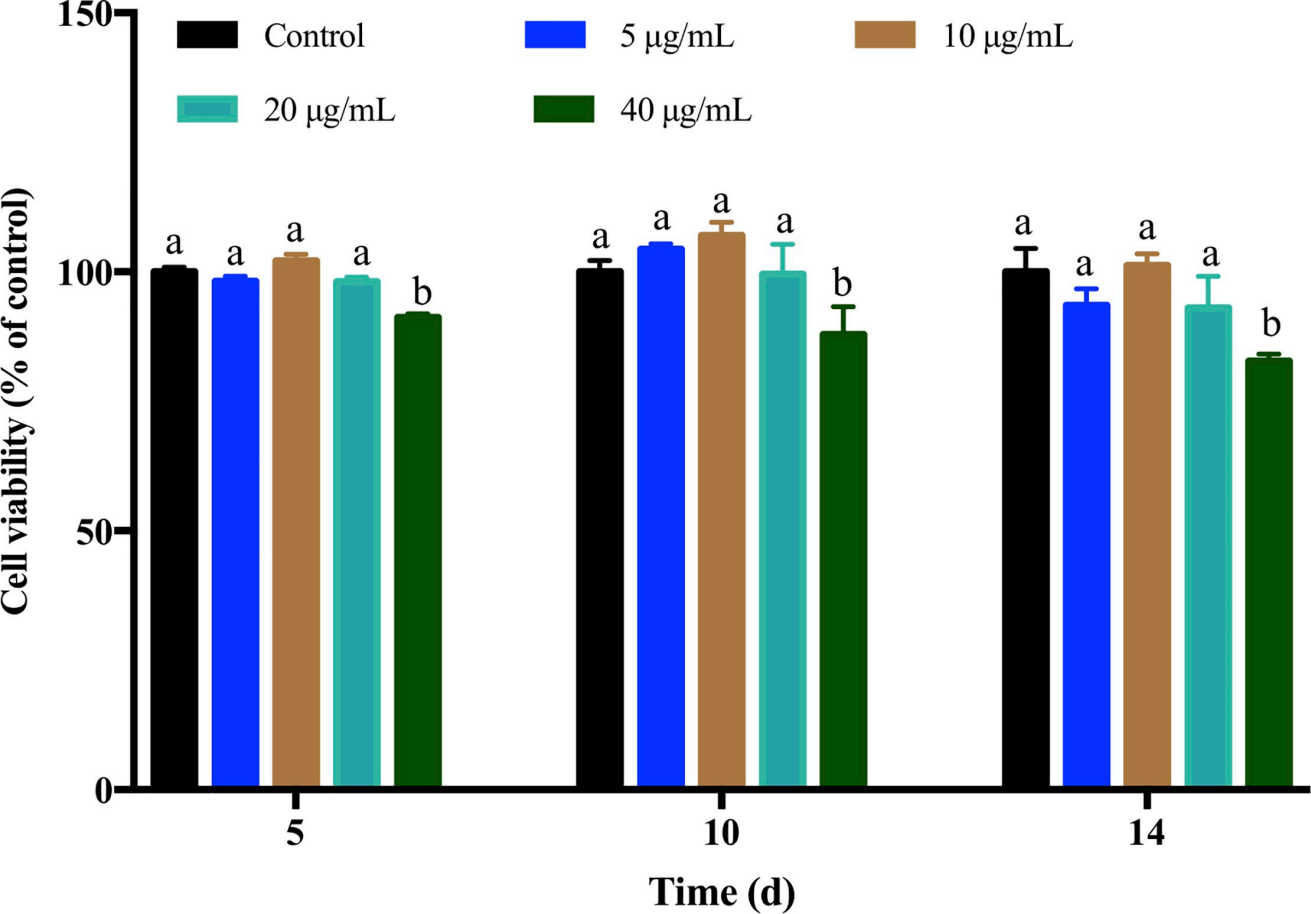
Effect of V-P complex on the cell viability. a, b Different letters indicate a significant difference (p< 0.05) within the graph.

The differentiation of preadipocytes leads to an increase in adipocyte number which is closely associated with obesity [10]. The growth of cells at different concentrations of the V-P complex are shown in Fig 1. It can be found that low concentrations of V-P complex (5, 10, 20 μg/mL) had no adverse effect on cell viability during differentiation. However, the cells treated with 40 μg/mL of V-P complex displayed a significant time-dependent reduction, the maximum inhibitory effect (17.25%) was observed at day 14 (Fig 1). These indicated that V-P complex with concentration less than 20 μg/mL had low cytotoxicity, which were in agreement with literature [23], in which VBP from sea squirt had low cell toxicity after differentiation in 3T3-L1 for 6 days. It has been demonstrated that organic vanadium compounds didn’t cause any gastro-intestinal discomfort or liver and kidney toxicity [29]. Therefore, the V-P complex with concentrations of 5, 10 and 20 μg/mL were selected for subsequent studies.

### Effect of V-P complex on lipid accumulation

**Fig 2:**
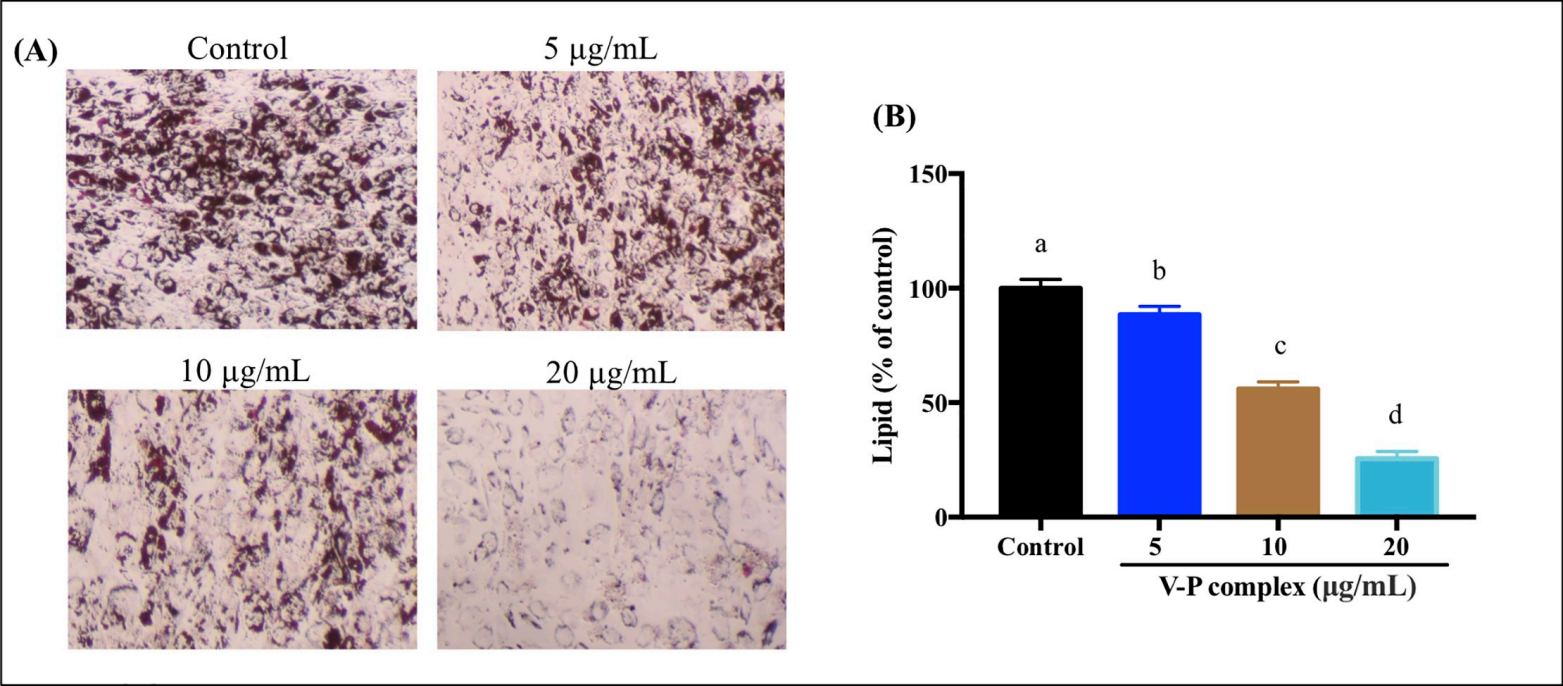
Effect of V-P complex on cellar lipid droplets. **(A)** Confluent human preadipocytes were induced to differentiate in DM containing different concentrations of V-P complex for 14 days **(B)** Oil red O were extracted and quantified by measuring the absorbance at 490 nm. All values present the mean ± SD (n = 5) of three independent experiments. a, b, c, d Different letters indicate a significant difference (p< 0.05) within the graph.

Our previous work showed that the synthetic V-P complex had high antidiabetic activity *in vitro* [24]. Here, the effect of different concentration of V-P complexes on the human preadipocyte differentiation was determined. Lipid contents in differentiated human preadipocytes at day 14 were monitored. It can be observed that V-P complex at the concentration of 5, 10 and 20 μg/mL decreased intracellular lipid accumulation compared to the control cells as revealed by microscopic observation following oil red O staining in differentiated human preadipocytes (Fig 2A). As shown by oil red O elution (Fig 2B), the V-P complex decreased lipid content in the differentiated human preadipocytes, in a dose-dependent manner at day 14. The V-P complex at 20 μg/mL can strongly inhibit the differentiation of human preadipocytes into mature adipocyte and decreased the lipid content by 74.47% (p<0.05). The reduction of lipid content observed in this work and previous studies might be attributed to the existence of vanadium in vanadium-protein compounds [10, 12]. These results were supported by Gunasinghe and Kim [23], in which the inorganic vanadium and VBP decreased the lipid content in 3T3-L1 differentiated cells at day 6. It has been demonstrated that lipid content and the extent of body fat accumulation are tightly associated with the occurrence and development of obesity [30]. Generally, lipid accumulation indicates the differentiation of preadipocyte into adipocyte and the oil droplets are used as a major marker of lipid accumulation [31]. In the present study, the decrease of lipid droplets caused by the V-P complex suggested that the V-P complex might involve in the process of human preadipocytes into adipocytes and block cellular lipid accumulation to exert the inhibitory effect on adipogenesis and then, possessed antiobesity activity.

### Effect of V-P complex on TG content

**Fig 3:**
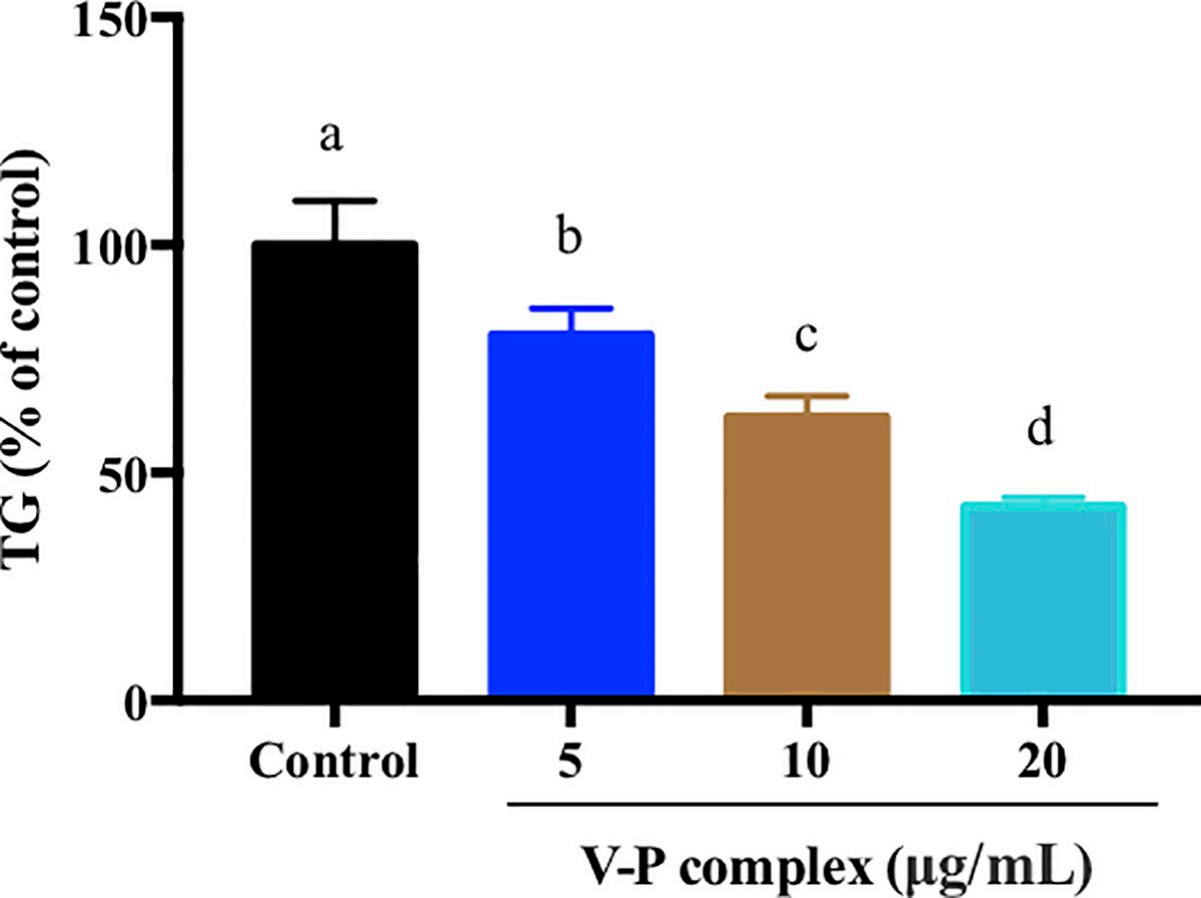
Effect of V-P complex on cellular triglyceride mass. All values present the mean ± SD (n = 5) of three independent experiments. a, b, c, d Different letters indicate a significant difference (p< 0.05) within the graph.

Generally, obesity is tightly related to adipocyte hypertrophy, and the size of the maximum fat cell was about twice higher than that of normal cells when the body mass was more than 170% to standard [32]. The enlargement of adipocyte size was attributed to the accumulation of TG in adipocytes [33]. To evaluate the potential inhibitory effects of the V-P complex on the size of human adipocytes, the TG content in differentiated human preadipocytes was determined using the triglyceride assay kit. A remarkable reduction of TG accumulation in differentiated human preadipocytes was observed at day 14 with the increase of V-P complex concentration (Fig 3). The TG content of human preadipocyte treated with 20 μg/mL of V-P complex decreased by 57.39% (p<0.05) compared to that of the control cells. Our results were agreement with previous study [34], in which VOdipic-Cl showed a dose-dependent reduction of TG content in 3T3-L1 adipocytes at day 8. Additionally, although the storage and release of TG play an essential role in maintaining energy balance, the excess amount of TG storage could lead to the development of obesity [10]. These indicated that V-P complex might execute antiobesity activity through suppressing TG accumulation.

### Effect of V-P complex on the protein expression of adipocyte specific genes in differentiated human preadipocytes

Obesity is the result of an increase in adipose tissue mass, which is caused not only by an increase in fat cell number because of adipocyte proliferation, but also by differentiation of pre- and post-confluent preadipocyte to adipocyte [28]. It is well known that a series of enzymes and adipogenic transcription factors such as PPARγ, C/EBPα and SREBP-1c play cross-regulation role in differentiation of preadipocytes into adipocyte, accumulation of lipid droplets in adipocytes and keeping adipocyte phenotype [28, 34]. The activation of PPARγ and C/EBPα can regulate terminal differentiation process through activating the downstream adipocyte-specific target genes such as FAS, lipoprotein lipase (LPL) and so on [35]. It has been demonstrated that FAS is a lipogenic enzyme and plays vital role in controlling fatty acid biosynthesis and TG storage in cytoplasmic [8], and the lipolytic enzyme LPL can limit lipolysis and triglyceride hydrolysis in the fat tissue [36].

**Fig 4:**
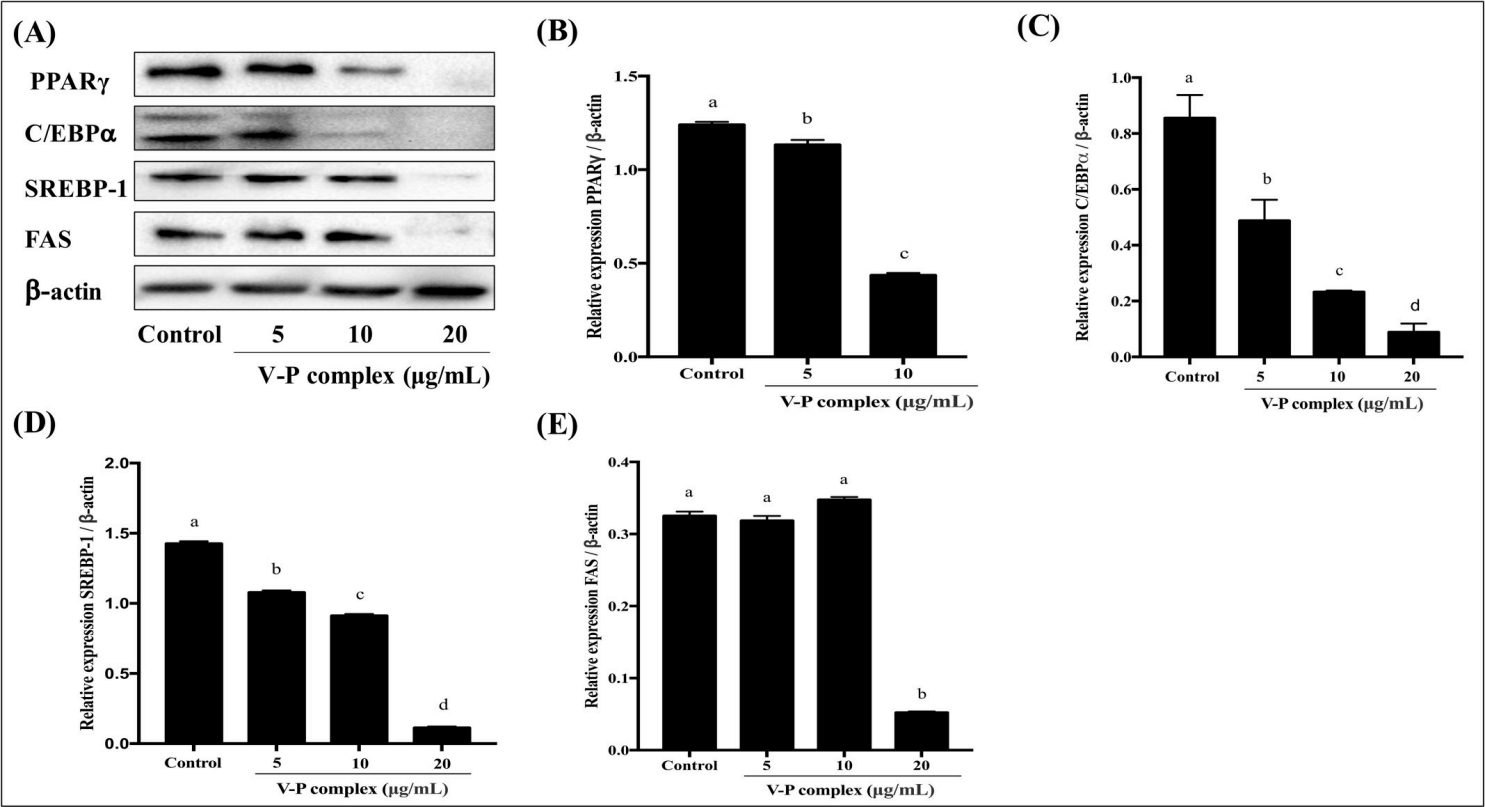
Effect of V-P complex on protein expression of adipocytes-related specific genes. a, b, c, d Different letters indicate a significant difference (p< 0.05) within the graph.

The fact that V-P complex decreased the number of differentiated human preadipocytes prompted us to investigate the influence of these vanadium compounds on protein expression of PPARγ, C/EBPα, SREBP-1 and FAS. Western blot analysis showed that the relative protein expressions in differentiated human preadipocytes remarkably reduced at 5, 10 and 20 μg/mL of V-P complex, from 8.60 to 100.00% for PPARγ, from 42.96 to 89.69% for C/EBPα and from 24.42 to 92.10% for SREBP-1, respectively (Fig 4). These results indicated that V-P complex had a potential in anti-adipogenesis. Additionally, FAS, a terminal marker of adipocyte differentiation, was also investigated. The relative protein expression of FAS in differentiated human preadipocytes was reduced by 89.69% at 20 μg/mL of V-P complex (Fig 4), further indicating that V-P complex had anti-adipogenic activity. A significant down-regulation of PPARγ, C/EBPα, SREBP-1 and FAS protein expressions in the present study were in agreement with the previous research [12, 23], in which the natural VBP showed inhibition effect on adipocyte differentiation by decreasing the protein expression of PPARγ, C/EBPα and FAS in 3T3-L1 adipocytes. It has been demonstrated that the inorganic vanadium AMV reduced the expression of PPARγ, C/EBPα, SREBP-1 and FAS, while increased the expression of LPL in 3T3-L1 mature cells at day 6 [23]. Additionally, previous studies have reported that male B6⋅BKS (D) *db*/*db* mice were treated with synthetic vanadyl complexes, the protein expression of PPARα/γ in adipose tissues was increased, revealed that vanadium compounds had potent insulin enhancement effect [37, 38]. In the present work, the inhibition of protein expression of PPARγ, C/EBPα and SREBP-1 caused by V-P complex implied that this V-P complex could prevent adipocyte differentiation through PPARγ, C/EBPα and SREBP-1 modulated adipogenesis mechanism which was related to FAS gene, one of the downstream adipocyte specific genes [39].

### Effect of V-P complex on the expression of adipocyte specific genes in Wnt/β-catenin pathway

Adipocyte differentiation is a complex process that the undifferentiated fibroblast-like preadipocytes differentiate into mature lipid-filled adipocytes [9]. It involves the integration of many adipogenic transcription factors and different signaling pathways such as Wnt/β-catenin, MAPK, AMPK and PI3K/AKT. Among these pathways, the Wnt/β-catenin has been confirmed as an important regulator of adipocyte differentiation [9]. Once the Wnt/β-catenin pathway was activated, cytoplasmic β-catenin was released and translocated to nucleus to regulate the expression of PPARγ and C/EBPα, in which high-level expression of β-catenin plays a central role in blocking adipogenesis [9, 12, 40]. Furthermore, the recent investigation showed that the Wnt signaling palyed an important role in metabolism and adipocyte biology in obesity and type 2 diabetes [9]. The previous study also suggested that the natural vanadium compound VBP inhibited 3T3-L1 preadipocyte differentiation through activation of Wnt/β-catenin pathway [12, 23].

**Fig 5:**
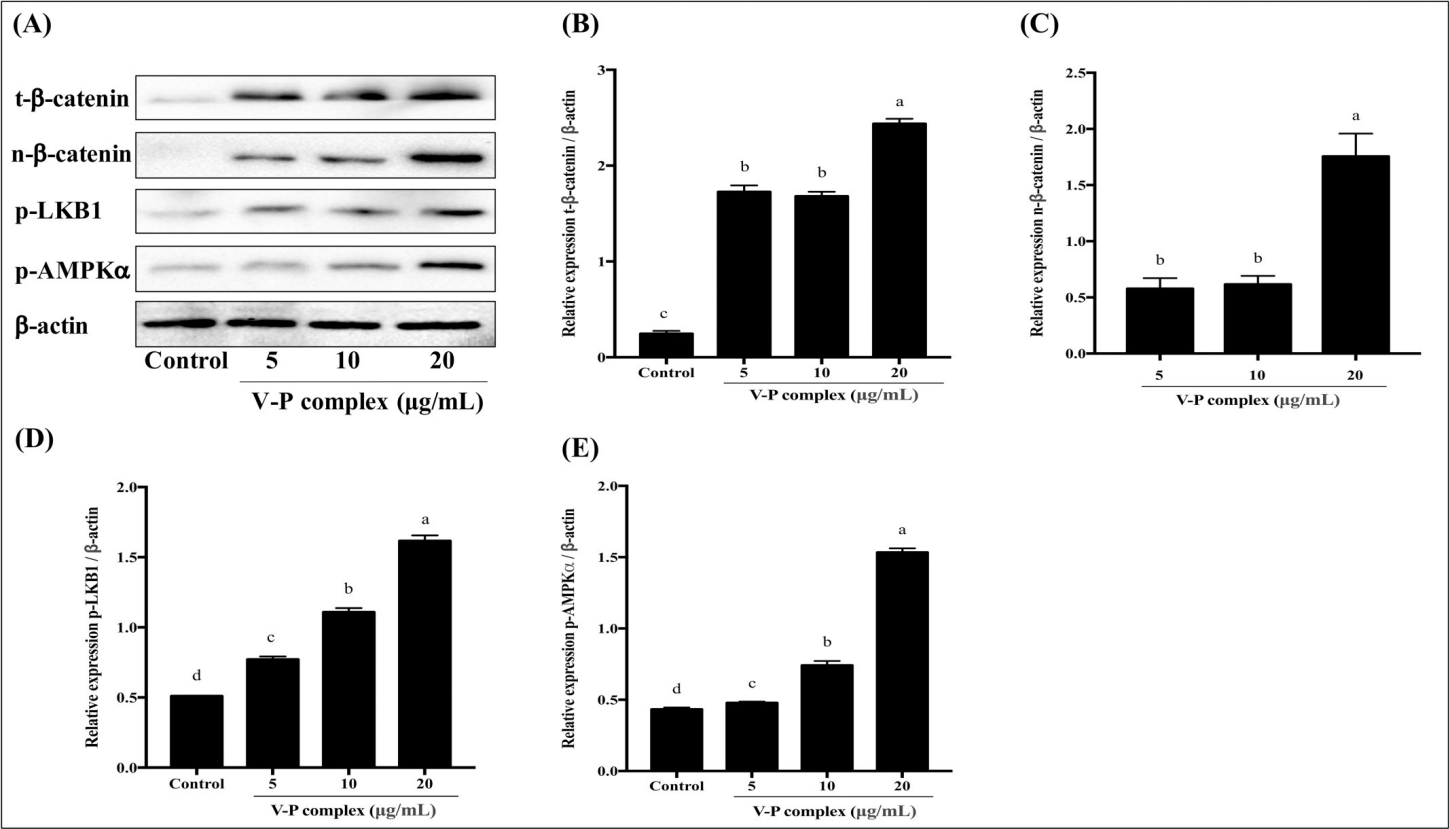
Effect of V-P complex on the protein expression. a, b, c, d Different letters indicate a significant difference (p< 0.05) within the graph.

**Fig 6:**
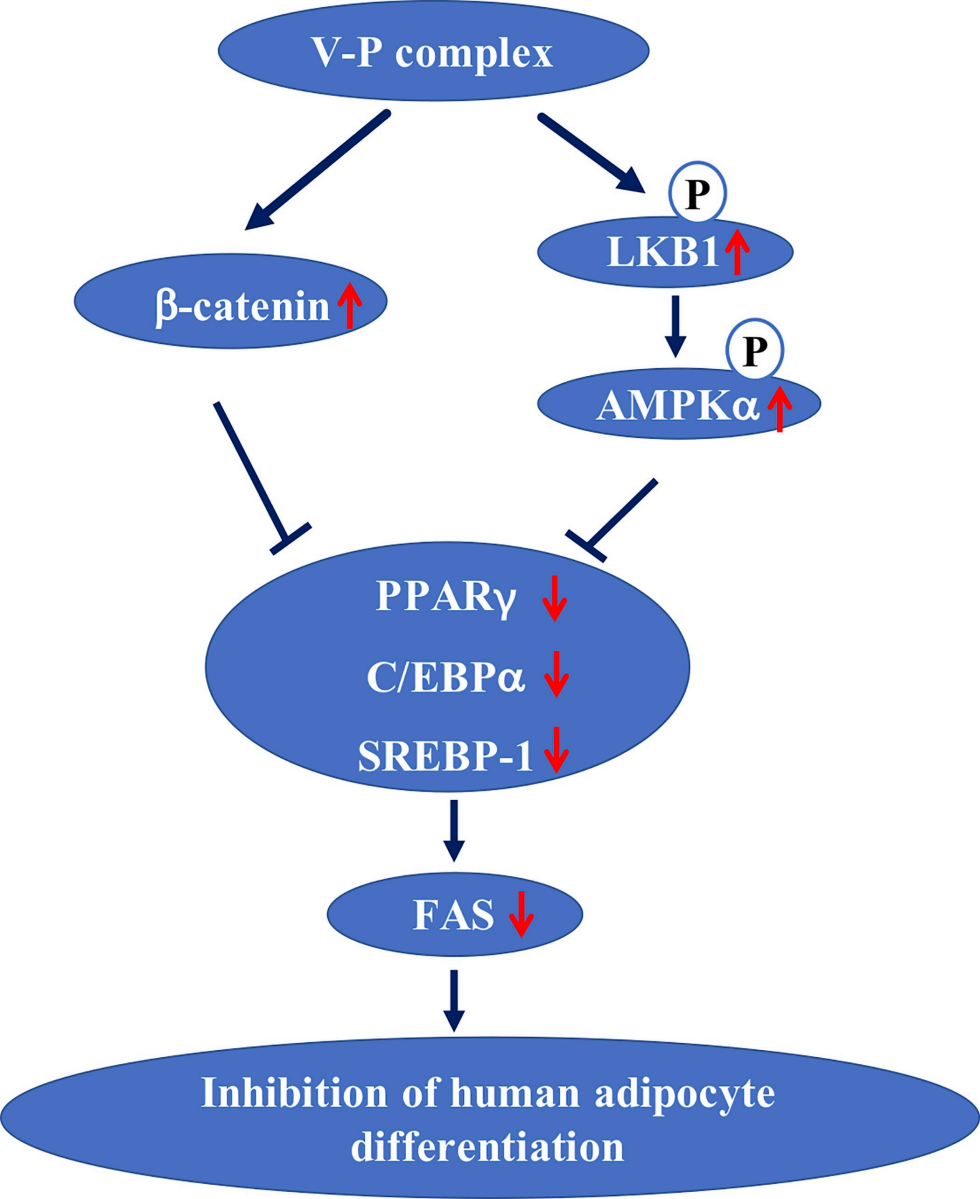
A hypothesis of the molecular mechanism of the inhibitory effect of V-P complex on the adipogenesis in human preadipocytes. Black arrow proposed pathway, red arrow indicated the effects, T inhibition.

In the present study, to determine whether synthetic V-P complex could regulate adipogenesis in human preadipocytes by activating Wnt/β-catenin pathway, the protein expression levels of t-β-catenin and n-β-catenin in differentiated human preadipocytes at different concentrations of V-P complex were monitored at day 14. It can be observed that the protein expression of t-β-catenin and n-β-catenin significantly increased in the V-P complex-treated human preadipocytes compared to control cells (Fig 5). The protein expressions of t-β-catenin at 5, 10 and 20 μg/mL of V-P complex respectively increased 7.0-, 6.8- and 9.9-fold, while n-β-catenin relative protein expressions severally increased to 0.58-, 0.62- and 1.75-fold comparing to the control cells (Fig 5). These results were in agreement with the results of Liu et al. [12], in which natural VBP from sea cucumber significantly increased the protein expressions of t-β-catenin and n-β-catenin. Both the synthetic V-P complex in the present study and natural VBP in the previous study appeared a similar influence on adipocyte differentiation, indicating that V-P complex could be used as a health supplement to replace natural VBP in the management of obesity. In addition, the β-catenin in Wnt/β-catenin pathway was initially known as Wnt effector molecule [9]. The Wnt signaling function was regarded as an adipogenic switch to suppress or initiate adipogenesis when it was turned on or off [40]. Recently, β-catenin was known as a transcriptional co-activator in Wnt/β-catenin pathway [41]. It has also been reported that the preadipocytes maintain in an undifferentiated state through activation of β-catenin, resulting in subsequent inhibition of two well-known key adipogenic transcription factors, PPARγ and C/EBPα [40, 41]. Therefore, our results indicated that the adipocyte differentiation by the V-P complex was attenuated through up-regulation of the β-catenin (Fig 6) [9].

### Effect of V-P complex on LKB1/AMPK pathway

The LKB1/AMPK pathway plays critical roles in adipocyte differentiation [4, 16, 42]. The AMPK is an essential regulator of adipocyte differentiation, and LKB1 is the upstream regulator of AMPK [16, 43, 44]. In the present study, to investigate whether V-P complex could modulate adipocyte differentiation through LKB1/AMPK pathway, human preadipocytes were treated with different concentrations of V-P complex during differentiation. It can be found that the protein levels of phosphorylated LKB1 and AMPKα were up-regulated by V-P complex and reached their maximum values at the 20 μg/mL (Fig 5). As shown in Fig 5, the V-P complex at 5, 10 and 20 μg/mL produced 1.5-, 2.2- and 3.5-fold increase in LKB1 phosphorylation and 1.1-, 1.7- and 3.5-fold increase in AMPK phosphorylation, respectively. These results were supported by Zhang et al. [10], in which VOdipic-Cl inhibited adipocyte differentiation through activating LKB1/AMPK pathway. It has been demonstrated that LKB1 existed in AMPK-activated protein kinase cascade is an upstream kinase in fat tissue [10, 42]. As an intracellular energy sensor, AMPK was sensitive to a series of metabolic and nutrient factors, resulting in regulating lipid metabolism [42]. Once activated LKB1/AMPK pathway, activated LKB1 can increase phosphorylation and activation of AMPK, which further modulated the expression of transcription factors of PPARγ, C/EBPα, SREBP-1 and inhibited adipocyte differentiation [34]. On the contrary, it has been demonstrated that the reduction in the protein expression of PPARγ, C/EBPα, FAS and FABP4 was diminished by blocking LKB1 or AMPK expression using LKB1 siRNA or AMPK siRNA [10]. Additionally, AMPK activation can also restrict fatty acid effluence, promote fatty acid oxidation and glucose transport, reduce cytokine secretion, triglyceride synthesis and lipogenesis in adipocytes, and then exert anti-adipocyte differentiation activity [13]. In this study, the V-P complex increased the protein expression of phosphorylated LKB1 and AMPKα (Fig 5). These indicated that V-P complex could inhibit adipocyte differentiation by activation of LKB1/AMPK signaling pathway in human preadipocytes (Fig 6), further confirming that V-P complex may have antiobesity activity and can be used as nutraceutical.

Actually, vanadium compound has attracted much attention in the past decade due to it is considered as one of the promising antioxidant and antidiabetic agents [26, 37, 38, 45, 46]. Several oxidovanadium complexes including [V(O)(oda)(H_2_O)_2_] ([oda = O(CH_2_COO^-^)_2_]) and [VO(IDA)phen]·2H_2_O were synthesized and characterized [45, 46]. Among them, the latter were found to scavenge superoxide free radicals (O_2_^·-^) and organic radicals (ABTS^·+^ and DPPH^·^) [46]. A novel aminophenol-derivatized nitrilotriacetic acid vanadyl complex, i.e., p-hydroxyl aminophenol derivative (VOphpada), was confirmed to possess high antioxidant activity and low cytotoxicity. It can reduce blood glucose level, enhance glucose tolerance and allay stress induced by hyperglycemia and hyperlipidemia [38]. The new N, N-dimethylphenylenediamine (DMPD)-derivatized nitrilotriacetic acid vanadyl complexes ([VO (dmada)]) were synthesized and appeared high antioxidant capacity and low cytotoxicity on HK-2 cells [37]. Additionally, a novel GQD-VO (p-dmada) complex based graphene quantum dots drug delivery systems was prepared and showed the profound effects on insulin enhancement and β-cell protection [26]. The studies mentioned above suggested that the synthesis of new vanadium compounds can be regarded as an important strategy to develop antidiabetic drugs.

It has been reported that the vanabin1 (12.5 kDa) and vanabin2 (15 kDa) formed by recombinant protein can bind 10 and 20 vanadium ions, respectively [29]. The difference was attributed to the different molecular size and amino acids composition [47]. Therefore, we prepared the V-P complex by the reaction of vanadyl sulfate and recombinant human sentrin-specific protease 8 (N-6His) on Sephacryl S-200 HR column. The V-P complex was fond to exhibit antioxidant and anti‐diabetic activity [24]. The molecular weight of 26.2 kDa of recombinant human sentrin-specific protease 8 (N-6His) contained about 212 amino acid residues, the ratio of amino acid residue to vanadium ions V would be 1: (>2). The vanadium concentration of the V-P complex was determined to 0.1 mg/kg [24]. Although the dialysis was carried out, the V-P complexes might be not a pure compound but a mixture of vanadium-protein complexes and none specific bound vanadyl iminodiacetate. Thus, we will adopt a more effective approach to remove none specific bound vanadyl iminodiacetates in the further study.

## Conclusions

In the present study, the effect of synthetic V-P complex on adipocyte differentiation in primary human preadipocytes was investigated. The data demonstrated that the V-P complex reduced lipid accumulation and TG content in a dose-dependent manner in differentiated human preadipocytes. It was observed that V-P complex was effective in suppressing adipogenesis by down-regulating the protein expression of adipogenic transcription factors PPARγ, C/EBPα, SREBP-1 as well as by down-regulating the protein expression of adipogenesis-related gene FAS. Moreover, V-P complex also inhibited adipocyte differentiation in primary human preadipocytes through activating Wnt/β-catenin and LKB1/AMPK signaling pathways. Additionally, our findings indicated that synthetic V-P complex could be used as the nutraceutical in management of diabetes or obesity.

## Supporting information

S1 Fig (PDF)
