# Supplementary figures and images for: Vanadium-protein complex inhibits human adipocyte differentiation through the activation of β-catenin and LKB1/AMPK signaling pathway
Source: PLoS One. 2020 Sep 24;15(9):e0239547. doi: 10.1371/journal.pone.0239547 (PMC7514027; doi:10.1371/journal.pone.0239547)

PPAR $\gamma$

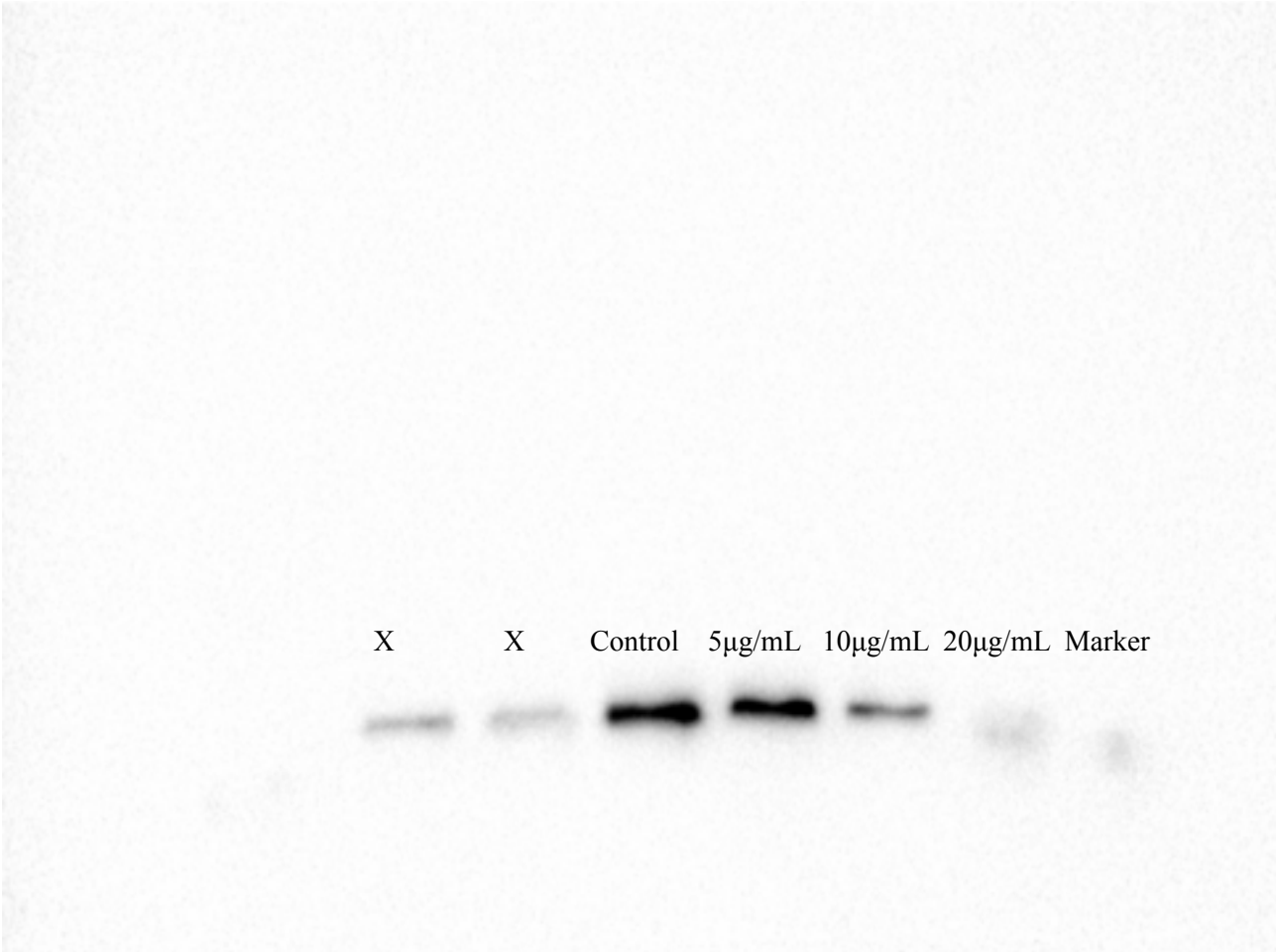

C/EBP $\alpha$

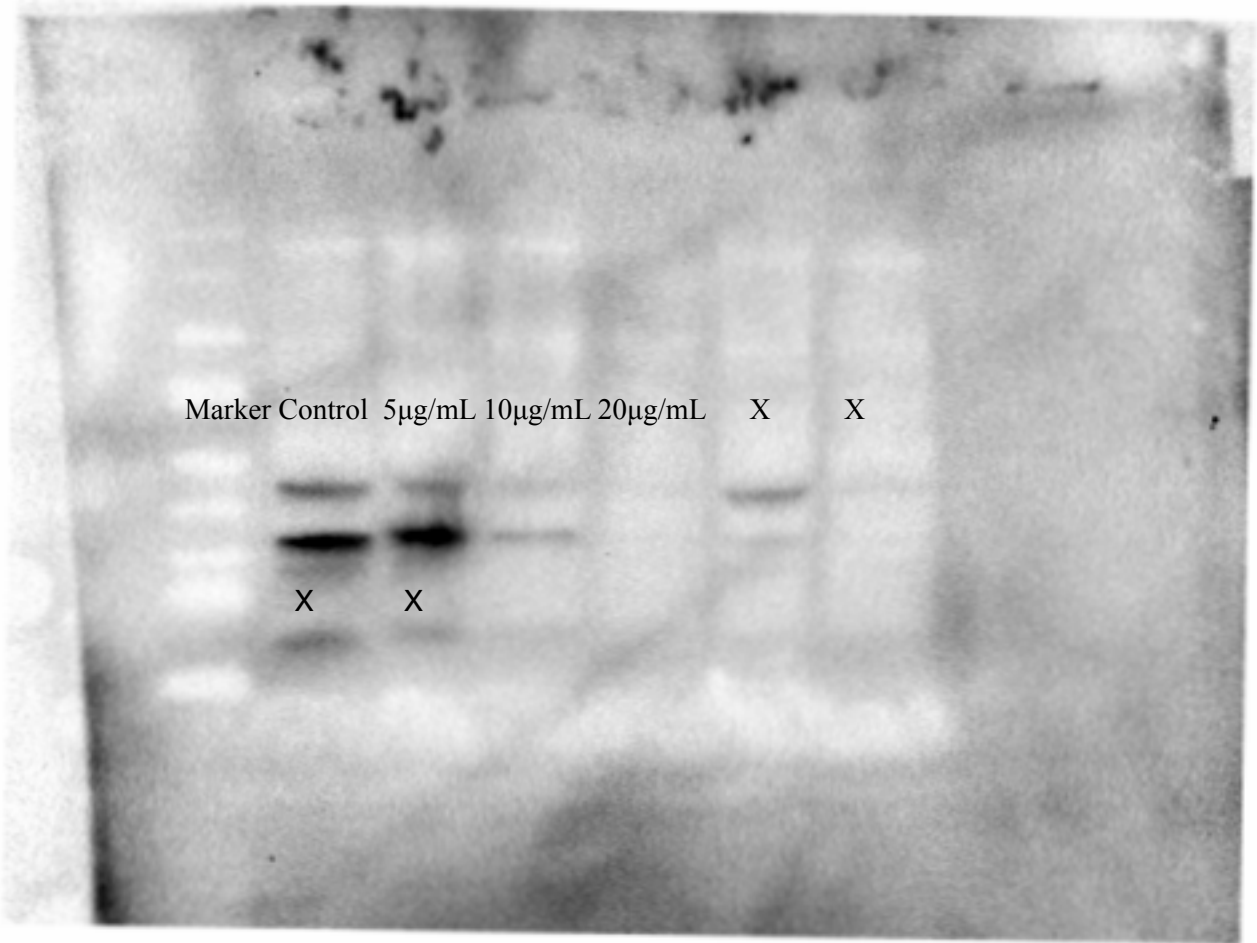

SREBP-1

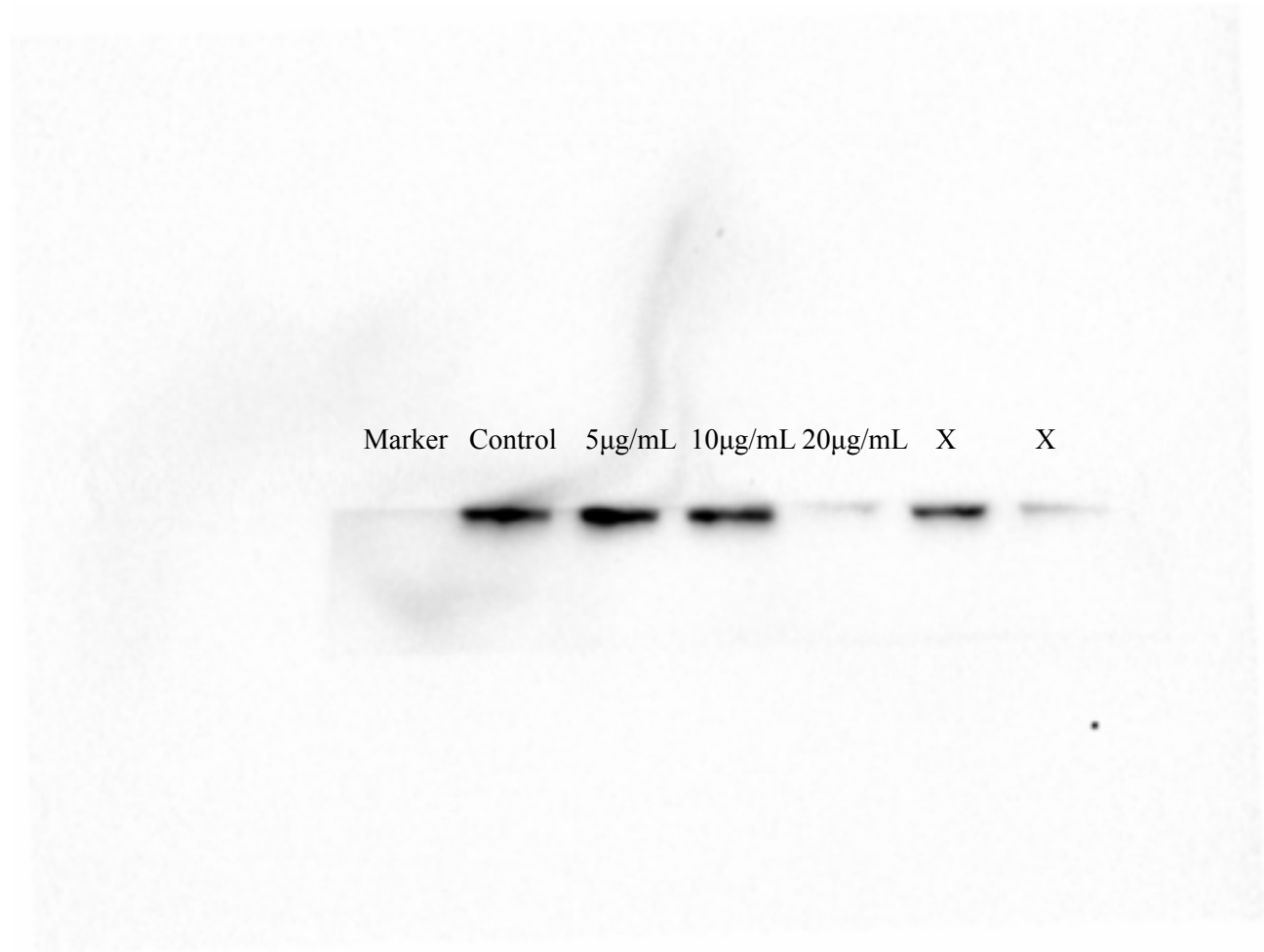

FAS

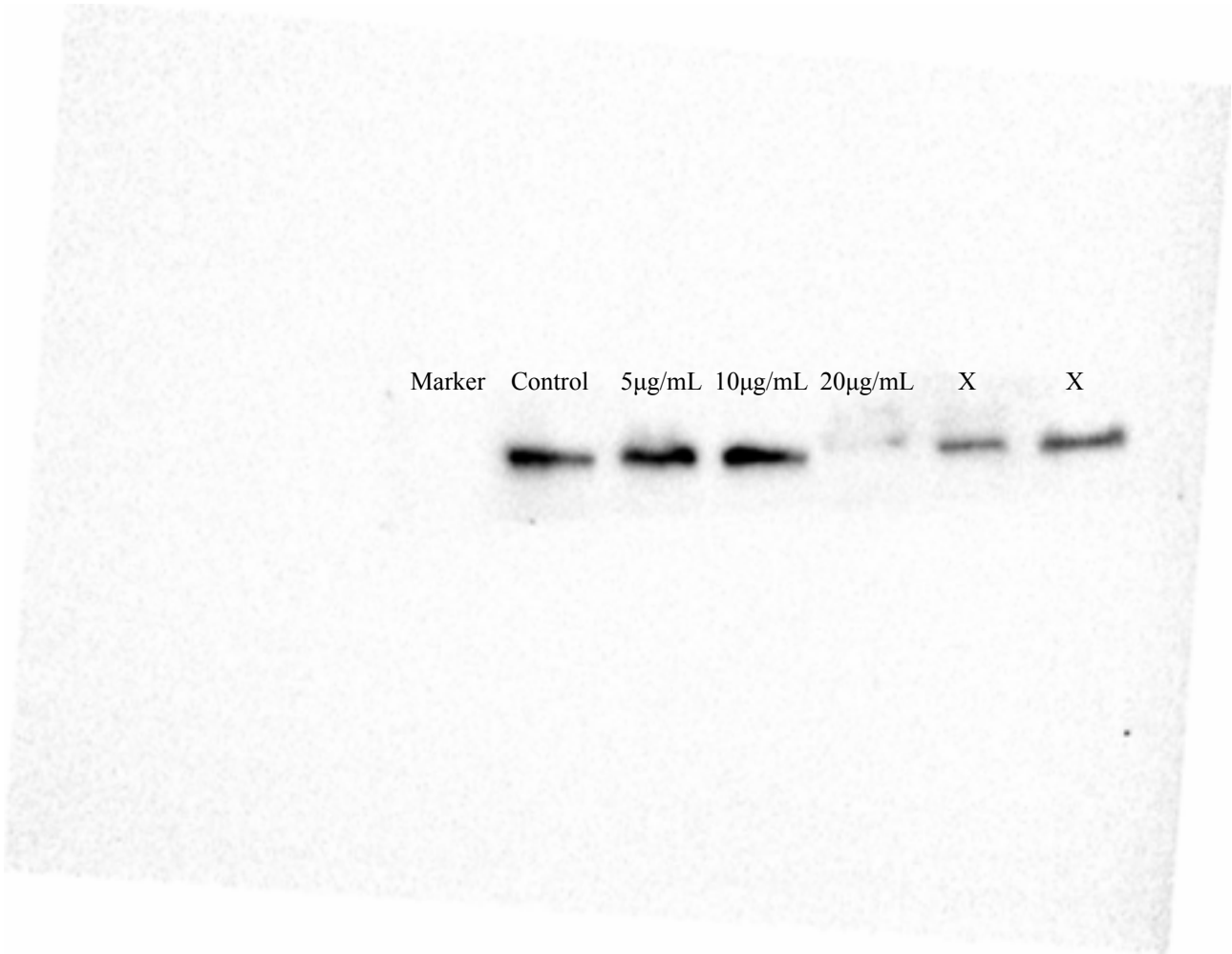

$\beta$ -actin

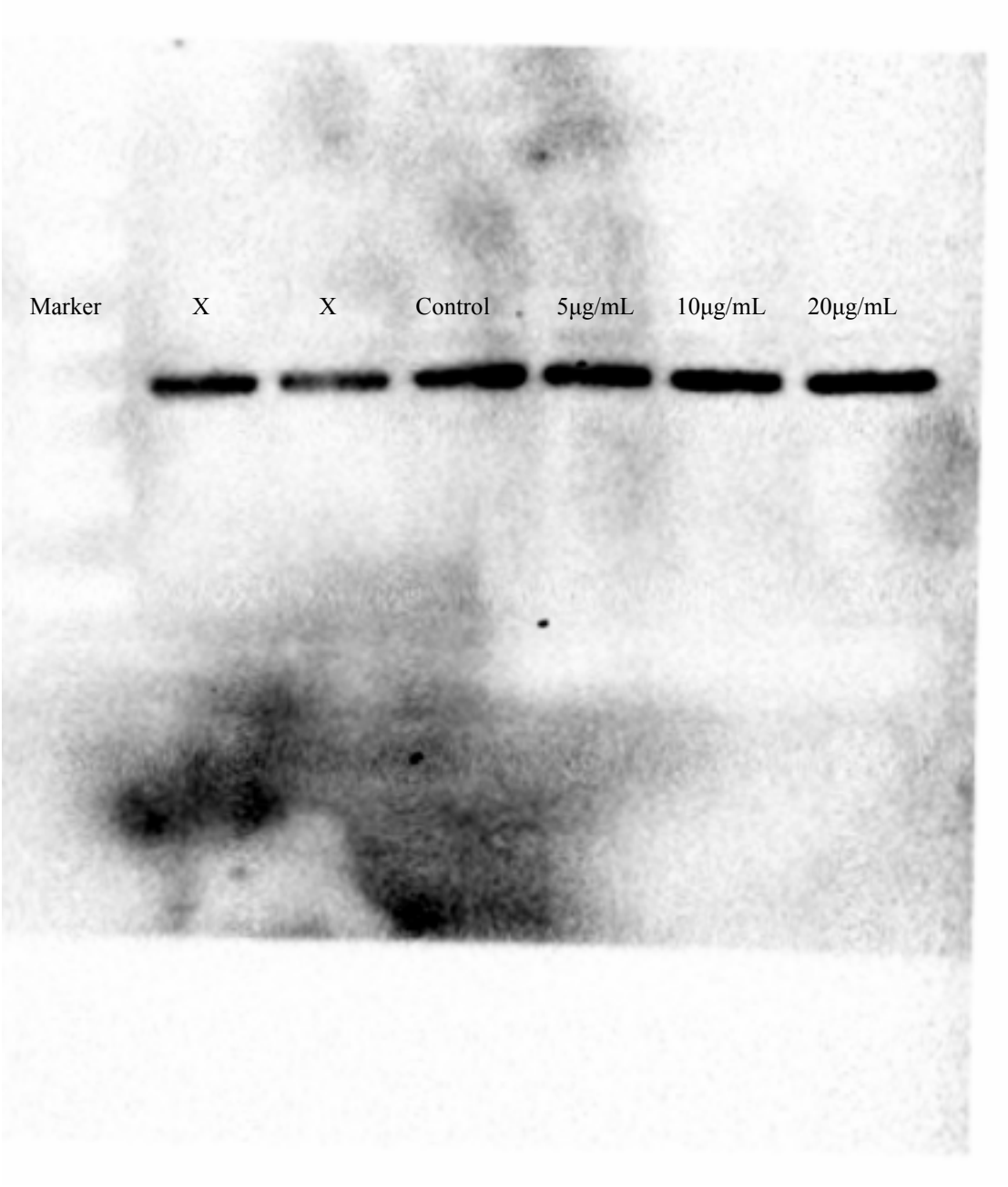

t-β-catenin

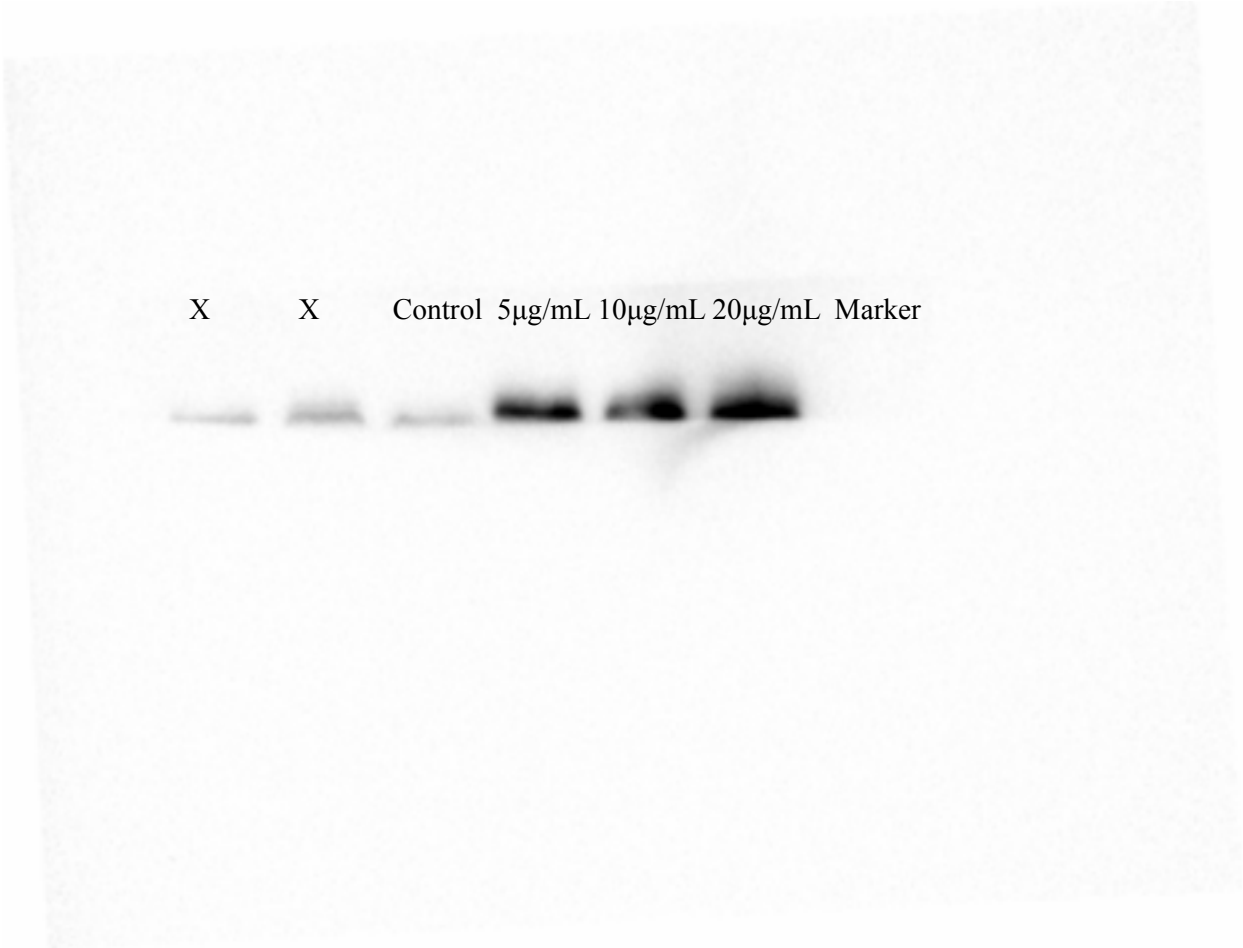

n-β-catenin

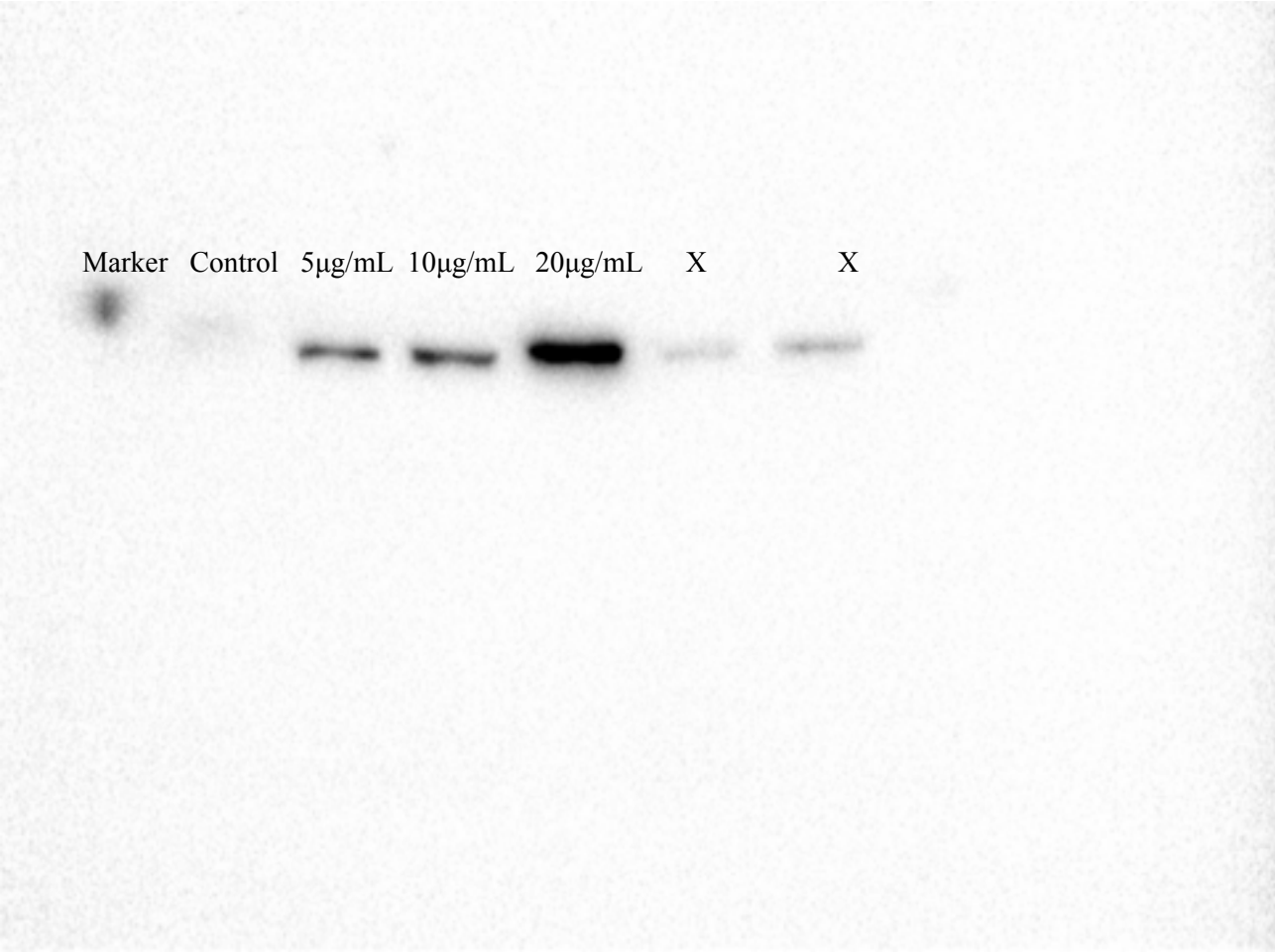

p-LKB1

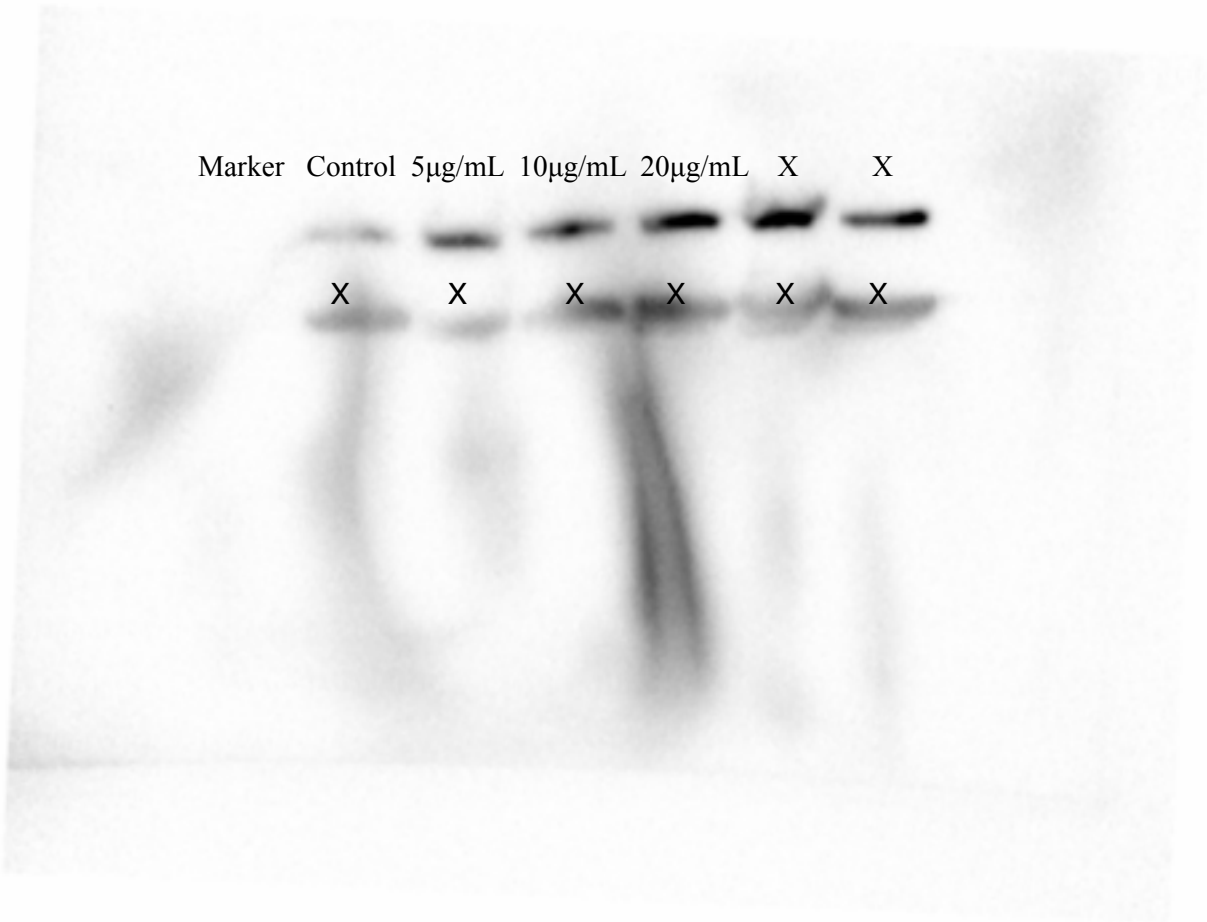

p-AMPK $\alpha$

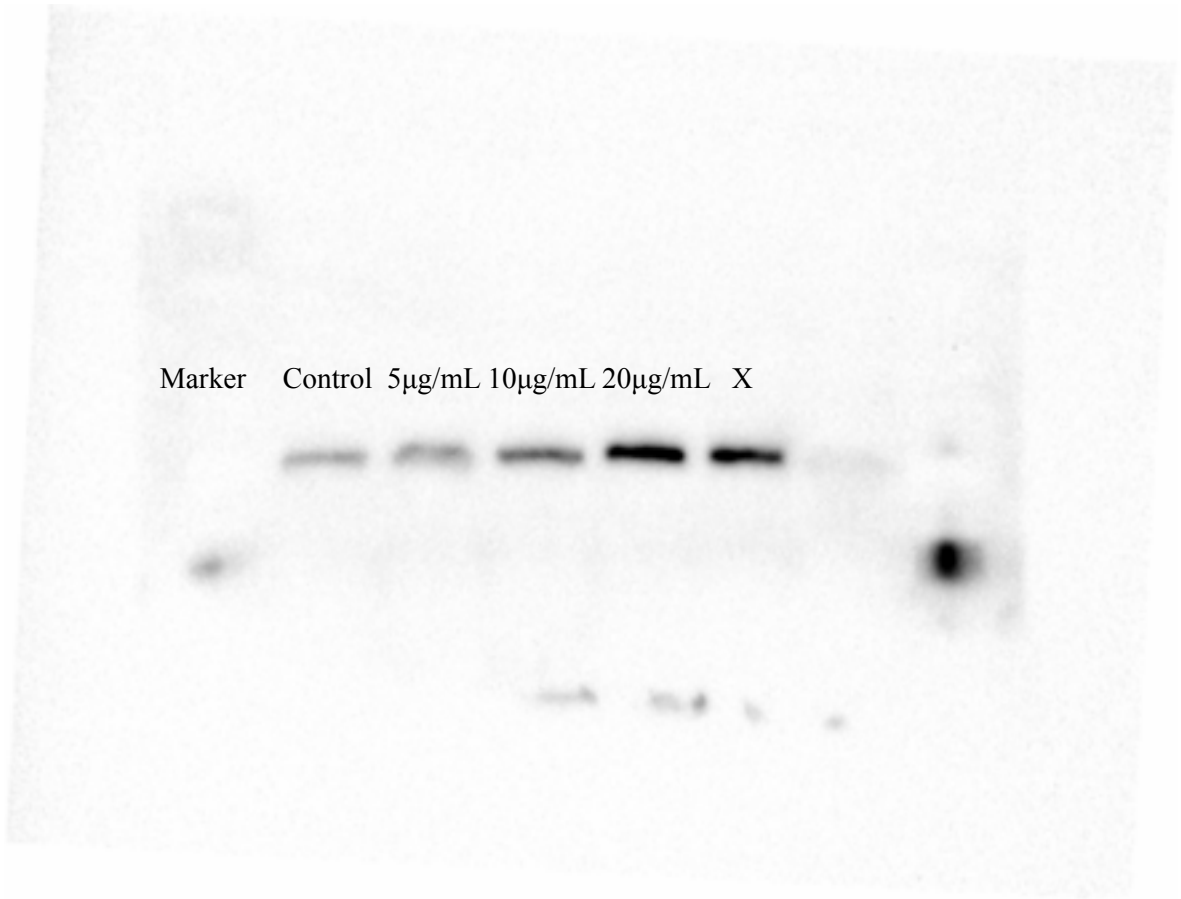

$\beta$ -actin

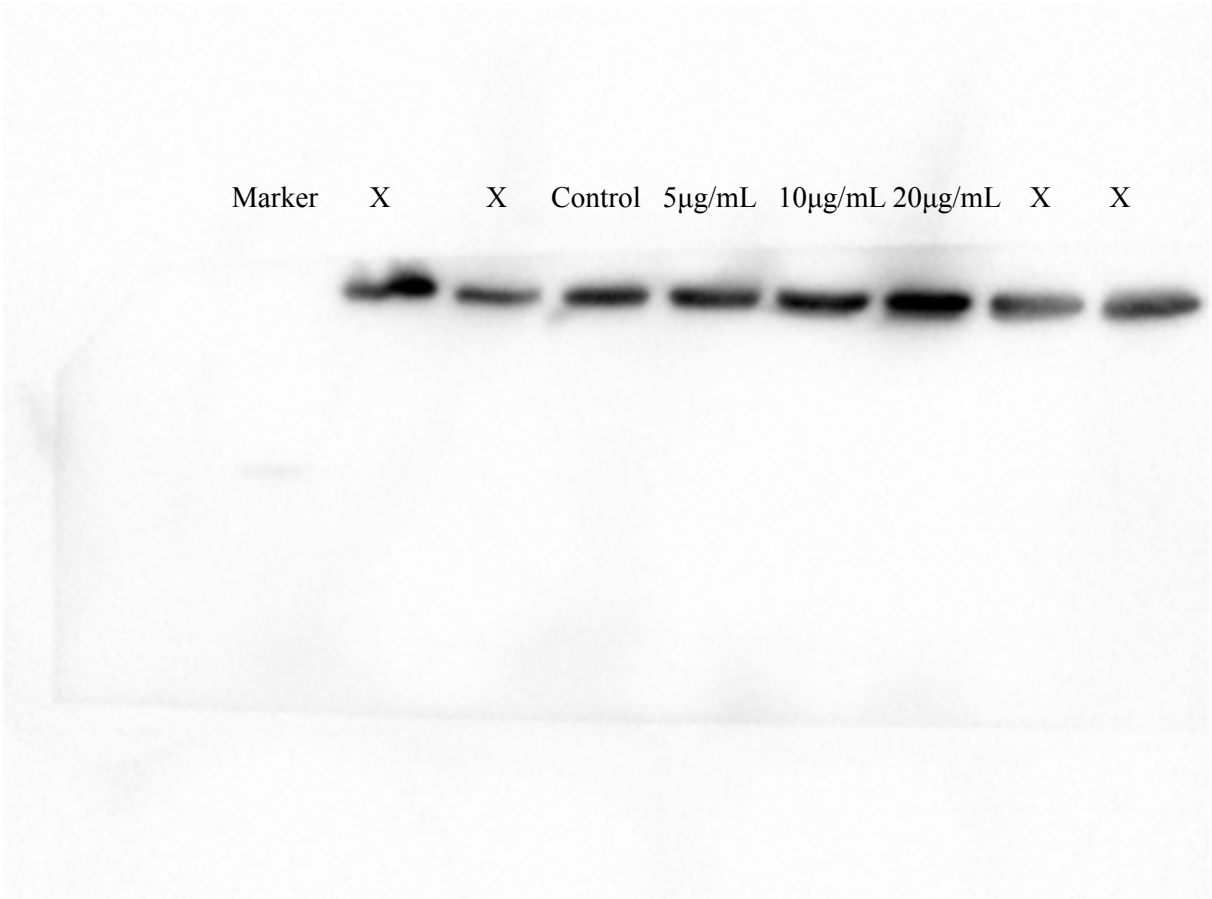

Supplement: S1 Fig — (PDF) [file pone.0239547.s001.pdf]
